# Synthesis and Characterization of Aminosilane Grafted Cellulose Nanocrystal Modified Formaldehyde-Free Decorative Paper and its CO_2_ Adsorption Capacity

**DOI:** 10.3390/polym11122021

**Published:** 2019-12-06

**Authors:** Wenkai Zhu, Meixiu Ji, Yang Zhang, Zhe Wang, Wei Chen, Yuanyuan Xue

**Affiliations:** College of Materials Science and Engineering, Nanjing Forestry University, Nanjing 210037, China; wenkai1992@njfu.edu.cn (W.Z.); meixiu0814@njfu.edu.cn (M.J.); zhewang1995@njfu.edu.cn (Z.W.); weichen1995@njfu.edu.cn (W.C.); yuanyuan0103@njfu.edu.cn (Y.X.)

**Keywords:** cellulose nanocrystal, amine modifcation, formaldehyde-free decorative paper, CO_2_ adsorption capacity

## Abstract

As one of the main consumables of interior decoration and furniture, decorative paper can be seen everywhere in the indoor space. However, because of its high content of formaldehyde, it has a certain threat to people’s health. Therefore, it is necessary to develop and study new formaldehyde-free decorative paper to meet the market demand. In this work, we have obtained formaldehyde-free decorative paper with high CO_2_ adsorption capacity. Here, cellulose nanocrystals (CNC) were prepared by hydrolyzing microcrystalline cellulose with sulfuric acid. The *N*-(2-aminoethyl) (3-amino-propyl) methyldimethoxysilane (AEAPMDS) was grafted onto the CNCs by liquid phase hydrothermal treatment, and the aqueous solution was substituted by tert-butanol to obtain aminated CNCs (AEAPMDS-CNCs). The as-prepared AEAPMDS-CNCs were applied to formaldehyde-free decorative paper by the spin-coating method. The effects of various parameters on the properties of synthetic materials were systematically studied, and the optimum reaction conditions were revealed. Moreover, the surface bond strength and abrasion resistance of modified formaldehyde-free decorative paper were investigated. The experimental results showed that AEAPMDS grafted successfully without destroying the basic morphology of the CNCs. The formaldehyde-free decorative paper coated with AEAPMDS-CNCs had high CO_2_ adsorption capacity and exhibited excellent performance of veneer to plywood. Therefore, laminating the prepared formaldehyde-free decorative paper onto indoor furniture can achieve the purpose of capturing indoor CO_2_ and have a highly potential use for the indoor decoration.

## 1. Introduction

Carbon dioxide (CO_2_), as a conventional component of the atmosphere and the emission of gases from human respiration, is harmless to human body under normal circumstances. However, under indoor conditions with dense crowds and poor ventilation, we will have various adverse reactions, such as breathlessness, dizziness, headache, and asthma. Therefore, it is increasingly important to capture and store indoor CO_2_ [1,2]. As the main veneer material of interior furniture, decorative paper is aminated to obtain CO_2_ adsorption properties, which has important practical significance. The methods of reducing indoor CO_2_ content by capture including solid adsorption, solvent absorption, membrane separation, ionic liquids, and so on [3,4,5]. Compared with the traditional liquid amine adsorption method, solid adsorption method has the advantages of less energy consumption, simple equipment, low cost, and less corrosion, which makes it able to be used in a broader field [6,7]. However, the recycling performance of the adsorbents will behave poorly and the possibility of large-scale application is less likely. Furthermore, it is very meaningful to develop new adsorbent. At present, a series of solid adsorbents mainly include zeolite, activated carbon, hydrotalcite, metal oxides, and solid amine adsorbents [8,9,10,11,12]. Amino-modified adsorbents have high selective adsorption capacity (they adsorb CO_2_ in the air instead of N_2_) and higher CO_2_ adsorption capacity [13,14]. Meanwhile, amine-modified adsorbents can be divided into impregnation and grafting methods according to their different synthesis methods. In the impregnation adsorption method, the interaction between amine and adsorbent is very weak, which leads to the poor thermal stability of the modified material and cannot be better used in the actual process. However, the grafting method introduces certain amino groups to form new chemical bonds through chemical reactions, which makes the modified material stable and expands its application scope [15].

Cellulose has characteristics of large reserves, wide distribution, renewability, and easy degradation, and it is considered as the future energy source in the world [16]. With the deepening of scientific research and the rise of nanotechnology, the demand for multi-functional composite materials is increasing day by day. Biomass cellulose nanocrystal (CNC) materials have become a hot research topic. Extracting CNCs from natural biomass materials and synthesizing products with specific functions has become a research trend in the field of fiber science and new materials [17,18]. Since there are a large number of bare hydroxyls, reducible and non-reducible end groups on the surface of CNCs, it is possible to modify the surface and manufacture functional materials [19]. In recent years, it has become a research focus to capture greenhouse gases such as CO_2_ by improving the adsorption performance of CNC through different modifications of surface functional groups. In addition, Maatar et al. synthesized nanofibrillar cellulose organogel (NFCo) by the freeze-drying method, and then modified the activated cellulose carbonyl group to form a new ester group, which was coupled with hexadecylamine [20]. It was found that the adsorption capacity of the modified NFCo aerogels was 10 times higher than the unmodified NFC. Furthermore they grafted nanofibrillarized cellulose with Fenton reagent, which increased the adsorption capacity of nanocellulose for heavy metals [21]. Additionally, the use of nanocellulose grafted with amino acids as a CO_2_ adsorbent has become one of the key research directions [22,23].

Water-based acrylic resin has the advantages of high gloss, weatherability, chemical resistance, and convenience. In recent years, it has shown a relatively rapid development rate [24,25]. Meanwhile, with the continuous promotion of the concept of green environmental protection, water-borne coatings with green environmental friendliness continue to get attention. Water-based acrylic resin with excellent comprehensive properties have been favored by the vast number of scientific research enthusiasts, making them widely used in paper-making, coating, and construction industries [26,27,28]. In this study, formaldehyde-free decorative paper was obtained by impregnating a decorative base paper with water-based acrylic resin. We obtained the CNC by the hydrolysis of microcrystalline cellulose (MCC) with sulfuric acid and then chemically grafted onto the CNC an amino modifier (AEAPMDS) to obtain AEAPMDS-CNC hydrogel. Furthermore, AEAPMDS-CNC was coated on formaldehyde-free decorative paper by the spin coating method to fabricate formaldehyde-free decorative paper with high adsorption to CO_2_. This study has broadened the application field of CNCs and applied aminoized nanocellulose to absorb CO_2_ in the decorative paper industry, which is conducive to the increase of industrial added value. Furthermore, it has important guiding significance for improving indoor air quality.

## 2. Materials and Methods

**Figure 1:**
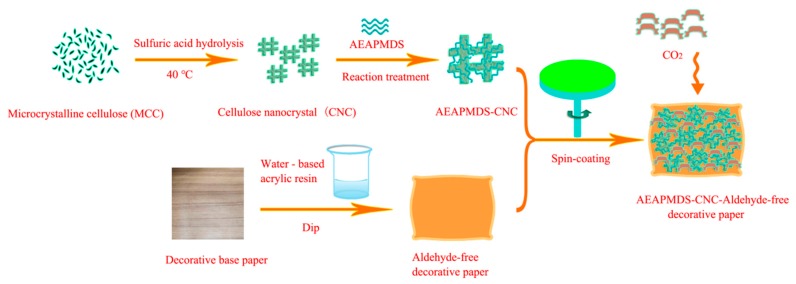
Schematic illustration of the preparation process for the AEAPMDS-CNC formaldehyde-free decorative paper.

A brief step in the preparation of AEAPMDS-CNC formaldehyde-free decorative paper is showed in Figure 1. Firstly, we used MCC as the raw material to obtain CNC by sulfuric acid hydrolysis. Secondly, amine-modified CNC was obtained by the liquid phase hydrothermal method. Finally, amine-modified CNC was coated on water-based acrylic resin-impregnated decorative base paper by the spin-coating method, and formaldehyde-free decorative paper with high CO_2_ adsorption property was obtained.

### 2.1. Materials

N-(2-aminoethyl)-3-amino-propylmethyldimethoxysilane (AEAPMDS, ≥ 99.7%) was supplied by Alfa Aesar (Heysham, UK). Microcrystalline cellulose (MCC, officinal class) was purchased from Shanghai Jinsui Bio-Technology Co., Ltd. (Shanghai, China). Decorative base paper (100 g/m^2^) and water-based acrylic resin (viscosity 37.6 mPa∙s; curing time of 2 min; concentration of the water-based acrylic resin is 59.57%) were supplied by Nanjing Bluewind New Material Science and Technology Co., Ltd. (Nanjing, China). Sulfuric acid (98%) and tert-butyl alcohol (99.7%) were purchased from Nanjing Chemical Reagent Co., Ltd. (Nanjing, China). All chemicals were of analytical grade and used without further purification. Distilled water was used in all of the experiments and was self-made.

### 2.2. Preparation of CNC

CNC can be obtained by hydrolysis of MCC with sulfuric acid. Briefly, 10 g of MCC was weighed and mixed with 63 wt% of sulfuric acid solution in a ratio of 1:10 (*m*/*v*), stirred magnetically for 1 h at 45 °C, then removed and diluted with 500 mL of distilled water to terminate the reaction. The solution was dialyzed and stopped at pH 7.0. The dialysis was processed by ultrasound for half an hour in an ultrasonic crusher with constant power of 2000 W.

### 2.3. Preparation of Amine-Modified CNC (AEAPMDS-CNC)

CNC with different mass fractions (1, 1.5, 2, 2.5, and 3 wt%) were used as raw materials, and the hydrogel was prepared after 5 min ultrasound in ice bath by the ultrasonic cell pulverizer. Then, AEAPMDS with different mass fractions (2, 4, 6, 8, and 10 wt%) were added. When the liquid phase was uniform, the samples were steamed and boiled in a water bath at different temperatures for a certain time (2, 4, 6, 8, 10, and 12 h). After standing at room temperature for 4 h, tert-butanol (75 wt%) was added for solvent replacement. Finally, the amine-modified CNC hydrogel samples were obtained.

### 2.4. Preparation of Formaldehyde-Free Decorative Paper Coated by AEAPMDS-CNC

The decorative base paper was soaked in water-based acrylic resin with a concentration of 20 wt% for 1 min, and then dried at 160 °C for 10 min to obtain formaldehyde-free decorative paper. The amine-modified CNCs prepared by the above experiments were applied to formaldehyde-free decorative paper by the spin-coating method, and then dried at room temperature to obtain AEAPMDS-CNC formaldehyde-free decorative paper. In order to investigate the physical properties of the modified formaldehyde-free decorative paper, we applied hot pressing technology to laminate it to formaldehyde-free plywood.

### 2.5. Characterizations

#### 2.5.1. Fourier Transform Infrared (FTIR) Spectra

The obtained CNCs and AEAPMDS-CNCs were mixed with potassium bromide in a mass fraction of 1:10, and then the samples were prepared in a tablet press. The infrared spectrum of the sample in the frequency range of 4000–500 cm^−1^ was measured by using an FTIR spectrometer (Nicolet 380, USA).

#### 2.5.2. Elemental Analysis

The nitrogen content in AEAPMDS-CNC under different preparation processes was tested using an elemental analyzer (Elementar vario EL III, Italy), each sample was tested in triplicate and averaged as the final test result.

#### 2.5.3. Thermogravimetric Analysis (TGA)

The thermal degradation behavior of the AEAPMDS-CNC samples were measured by a thermal gravimetric analyzer (Pyris 1 TGA, Perkin-Elmer Cetus Instruments, Norwalk, CT, USA). The 5 mg sample was weighed as the temperature ranged from 35 to 600 °C, with a heating rate at 20 °C/min. Nitrogen was flowed into the sample at 20 mL/min to prevent oxidation.

#### 2.5.4. Scanning Electron Microscopy (SEM) Micrograph

The morphologies or structures of the CNC and AEAPMDS-CNC samples were investigated by using a scanning electron microscope (SEM, Hitachi, Japan) at an operating voltage of 30 kV. Before the measurement, an appropriate amount of the samples were pasted on the sample table with double-sided adhesive tape, and observed after spraying with gold with the sputtering instrument.

#### 2.5.5. CO_2_ Adsorption Performance Test

CO_2_ adsorption and desorption at atmospheric pressure was measured by physical adsorption instrument (ASAP-2020, UAS). Before the test, samples in the adsorption bed were vacuum to −90 kPa for 30 min to eliminate water and CO_2_. During the adsorption process, the sample was placed on the adsorption bed and CO_2_ was charged to increase the pressure. The adsorption experiments were carried out for about 1 h under the condition of sealed adsorbent bed. The initial pressure was controlled within 0.5 Mpa, and the temperature was indoor temperature (25 °C). The initial pressure and the value of pressure in the airtight device after 60 min were recorded. Since the room temperature pressure is not too large, CO_2_ can be regarded as an ideal gas. The calculation formula is as follows:

(1) $$q=\left(\frac{P1V}{RT1}-\frac{P2V}{RT2}\right)/m$$

where *P*_1_ and *P*_2_ are the pressure values before and after adsorption, respectively. *V* represents the volume of adsorption bed. *T*_1_ and *T*_2_ indicate the temperature before and after adsorption. *R* represents the gas constants before and after adsorption. m represents the quality of formaldehyde-free decorative paper in the adsorption bed.

The regenerability test of the AEAPMDS-CNC formaldehyde-free decorative paper was performed by the above method and repeated for 10 cycles.

#### 2.5.6. Surface Bond Strength and Abrasion Resistance Test 

The surface bond strength of AEAPMDS-CNC formaldehyde-free decorative paper was tested by a universal ability test machine (CMT-4204, MTS, Shenzhen, China). The abrasion resistance of formaldehyde-free decorative paper coated with AEAPMDS-CNC was tested by an abrasion tester (JM-V, Tianjin World Expo Weiye Chemical Glass Instrument Co., Ltd., Tianjing, China).

## 3. Results and Discussion

### 3.1. Mechanism Analysis of Amine-Modified CNCs and CO_2_ Adsorption

**Figure 2:**
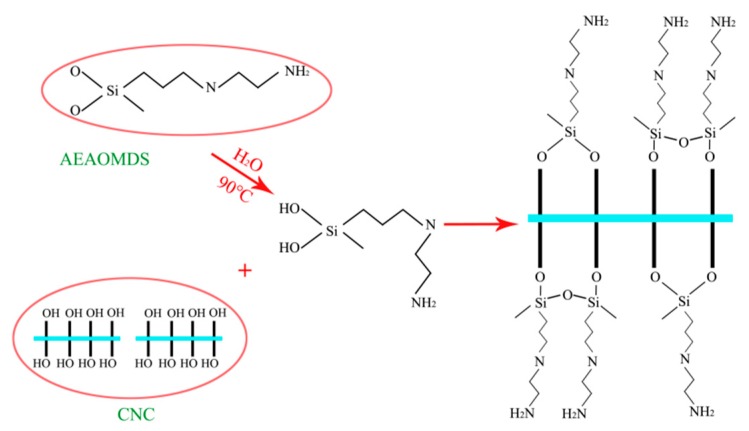
Schematic diagram of modification mechanism on CNC by AEAPMDS.

The reaction mechanism of amination modification of CNCs is shown in Figure 2. It can be sen from Figure 2 that the amine-modified CNCs mainly consisted of two parts: firstly, the amino silane AEAPMDS was hydrolyzed to produce amino silanol, and then the hydroxyl in the amino silanol was condensed with the active hydroxyl in the CNC molecule to formed a polymer with amino group. Additionally, because the condensation reaction of AEAPMDS-CNC is reversible, the yield of AEAPMDS-CNC was affected by time, temperature, dosage of the modifier, and concentration of CNC [29].

After the amination modification of AEAPMDS, CNC can achieve CO_2_ adsorption through the structure of the material surface and organic amine grafted. The reaction between organic ammonia and CO_2_ was essentially a reaction of weak base (ammonia) and weak acid. Primary and secondary amines reacted with CO_2_ to form carbamate, in which H atoms on the carboxyl group can form hydrogen bonds with adjacent amino groups, thus forming a stable chemical adsorption state. The adsorption mechanism is as follows [30,31]:

(2) $$\begin{array}{l} {CO}_{2}+\;{2RNH}_{2}\;\to {\;RNHCOO}^{-}\;+{\;{RNH}_{3}}^{+}\\ {CO}_{2}\;+\;2R_{2}NH\;\to {\;R_{2}NHCOO}^{-}+{R_{2}{NH}_{2}}^{+}\\ {CO}_{2}+R_{2}NH+R^{\prime }{NH}_{2}\;\to {\;R_{2}NCOO}^{-}+{R^{\prime }{NH}_{3}}^{+} \end{array}$$

### 3.2. Analysis of Nitrogen Content and Optimum Preparation Technology

**Figure 3:**
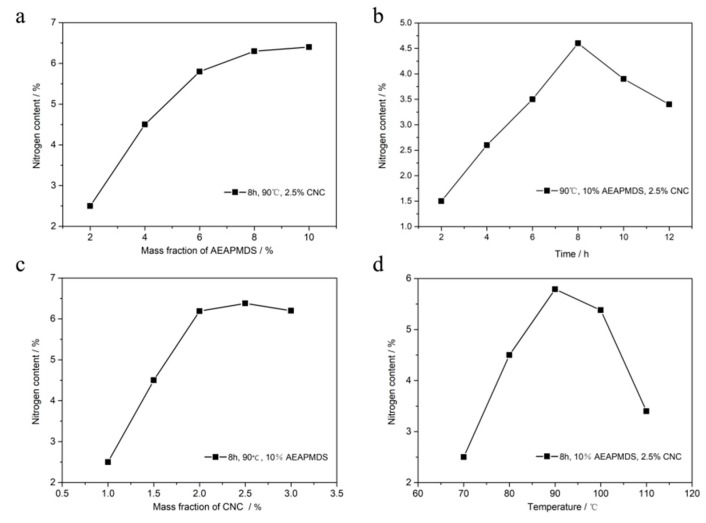
Effect of the mass fraction of AEAPMDS, reaction time, the mass fraction of CNC, and reaction temperature on N% of AEAPMDS-CNC. (**a**) Mass fraction of AEAPMDS; (**b**) reaction time; (**c**) mass fraction of CNC; (**d**) reaction temperature.

According to the above mechanism of CO_2_ adsorption by aminated CNC, we can know that the amino components of adsorbents play an important role in the process of CO_2_ adsorption. In other words, the mass fraction of amino group in AEAPMDS-CNC played an important role in the adsorption performance of CO_2_, and the content of nitrogen element directly reflects the mass fraction of amino group. Therefore, it is necessary to investigate the influence of the modification conditions on the nitrogen content in AEAPMDS-CNC [32]. Figure 3 shows the variety of nitrogen content in AEAPMDS-CNC under different reaction conditions (addition amount of AEAPMDS, reaction time, CNC content, and reaction temperature). Figure 3a shows the effect of modifier dosage on nitrogen content in AEAPMDS-CNC. It can be seen from Figure 3a that the content of nitrogen increases first and then tends to be stable with the addition of AEAPMDS. Since there is a reversible reaction between CNC and AEAPMDS in the reaction process, when the mass fraction of AEAPMDS was low, there were too many CNCs in the reaction solution and more active hydroxyl groups on the surface, so it was easier for aminosilanol to react with hydroxyl groups on the surface of CNC. Moreover, the content of AEAPMDS was limited, and the amount of AEAPMDS-CNC generated was relatively small. The direction of reverse reaction was weak, and the inhibition effect of reverse reaction on the forward reaction was also weak, so that the reaction can be converted into AEAPMDS-CNC compound with a higher limit when the mass fraction of AEAPMDS was low. Meanwhile, due to the limited active hydroxyl groups on CNC surface, excessive mass fraction of AEAPMDS had little effect on the conversion of reactants, so the rate of the increased nitrogen content tends to be stable.

The effect of reaction time on nitrogen content in amino CNC is shown in Figure 3b. When the reaction time was less than 8 h, the nitrogen content of the aminated CNC increased rapidly with the prolongation of reaction time. In this process, due to the large amount of reactants in the reaction solution, the reaction was carried out in the direction of forward reaction, and the rate of the forward reaction was significantly higher than that of the reverse reaction. As a result, the amount of the AEAPMDS-CNC compound generated by the forward reaction continued to increase. When the reaction time reached 8 h, the nitrogen content of the aminated CNC reached the maximum value. When the reaction time exceeds 8 h, the reaction efficiency gradually slowed down and the grafted rate decreased with the extension of the reaction time. This was because the condensation reaction reached saturation. After the full hydrolysis of AEAPMDS and the completed reaction of CNC, the continuous increase of the reaction time will lead to the reverse reaction, which will damage the amino bond grafted to the surface of cellulose and affect the graft rate of amine-modified CNC [33].

Figure 3c shows the effect of CNC mass fraction on the nitrogen content in the AEAPMDS-CNC. It can be seen from the figure that the nitrogen content of the modified AEAPMDS increased gradually with the addition of the CNC mass fraction. This is because the active hydroxyl group (–OH) on the surface of CNC in solution increased with the enlargement of the mass fraction of CNC. Increasing the amount of reactants made the reversible reaction moved towards the direction of generating AEAPMDS-CNC compound. Therefore, the increased amount of CNC in a certain range was helpful to increase the amount of active hydroxyl groups on the surface of CNC, thus increasing the content of nitrogen. This is consistent with the experimental results in Figure 3a. Additionally, when the mass fraction of CNC was 2.5 wt%, the amination effect of AEAPMDS was the most significant.

Figure 3d shows the effect of reaction temperature on nitrogen content in AEAPMDS-CNC. The results showed that the mass fraction of nitrogen increased firstly and then decreased with the increase in temperature. When the reaction temperature was lower than 90 °C, with the promotion of the temperature, the hydrolysis of AEAPMDS to aminosilicol reacted with CNC, and the amination modification efficiency increased with the rise of temperature. When the temperature was higher than 90 °C, the violent movement of water molecules reduced the content of water molecules in the reaction system, which has a counter-effect on the hydrolysis of amino silane, which was not conducive to the condensation reaction [34]. Thus, the optimum reaction temperature of aminated CNC was 90 °C.

### 3.3. Analysis of FTIR

**Figure 4:**
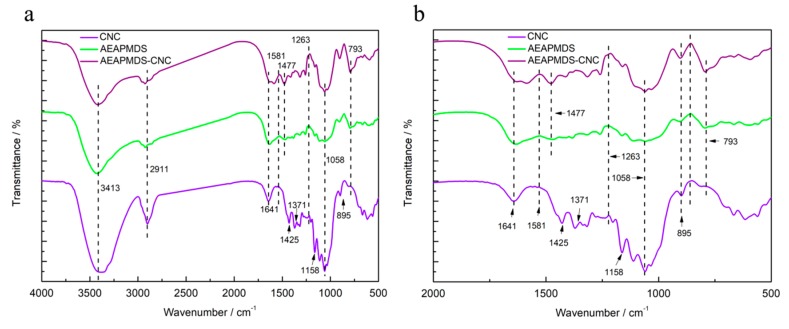
The FTIR spectra of the CNC, AEAPMDS, and AEAPMDS-CNC in the wave number range: 4000–600 cm^−1^ (**a**) and 1800–600 cm^−1^ (**b**).

In this study, FTIR spectroscopy was used to compare the chemical composition of CNC before and after modification. The infrared spectra of CNC and AEAPMDS-CNC are shown in Figure 4 and reflect the typical characteristic cellulose absorption peaks. The infrared spectra of CNC showed that the wider absorption peak near 3413 cm^−1^ was the O–H stretching vibration peak of CNC molecules adsorbing water. The absorption peak at 2911cm^−1^ was C–H stretching vibration absorption peak of methylene cellulose molecule. The peaks at 1425 cm^−1^ and 1058 cm^−1^ were CH_2_ shear vibration and stretch vibration peaks between the C–O of the carbon atom sixth in CNC, respectively [35,36]. The bending vibration peak of C–H was at 1371cm^−1^. The peaks at 1158 cm^−1^ and 895 cm^−1^ were the asymmetrical C–O–C stretching vibration [37]. The above peaks appeared in the infrared spectra of AEAPMDS-CNC, which indicated that the basic structure of CNC was not damaged after amination modification. In the unmodified CNC infrared spectra, the bending vibration peak of –OH appeared near 1641 cm^−1^, but weakened after added AEAPMDS. It may be that the AEAPMDS was easier to bind –OH in CNC than water, which reduced the amount of hydrogen bond between the CNC and water, thus, the peak appeared to weaken. According to the infrared spectra of the AEAPMDS-CNC, it can be seen that there was an obvious stretching vibration peak of –NH_2_ near 1581cm^−1^ [38]. In the AEAPMDS infrared spectra, the stretching vibration peak of –NH_2_ was not obvious, and it did not exist in the CNC. In the infrared spectrum of the AEAPMDS-CNC, the characteristic peak of –NH appeared at the peak of 1477 cm^−1^, C–Si telescopic vibration appeared at the peak of 1263 cm^−1^, and Si–O telescopic vibration appeared at the peak of 793 cm^−1^ [7,39]. The emergence of these new peaks proved that the AEAPMDS was successfully grafted onto the CNC.

### 3.4. Analysis of Thermal Stability

**Figure 5:**
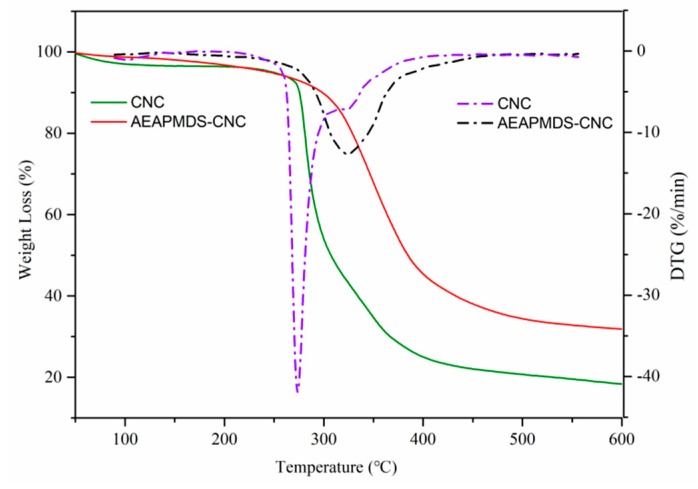
TGA and DTG spectra of the CNC before and after modification of AEAPMDS.

The thermal stability of the AEAPMDS-CNC with CO_2_ adsorption performance was investigated by thermogravimetric analysis (TGA) and derivative thermogravimetry (DTG). Figure 5 shows the TGA and DTG curves of CNC before and after the modification under certain heating rate and nitrogen atmosphere. According to the TG curve in the figure, we know that both CNC and AEAPMDS-CNC showed one-step thermal degradation. A very small amount of weightlessness occurred between 20 °C and 200 °C, which may be caused by the evaporation of the physically adsorbed water in the sample [40]. There was thermal degradation between 270 °C and 450 °C and the degradation rate of CNC was higher than that of AEAPMDS-CNC. Therefore, we know that the thermal stability of CNC was improved after amino modification. This may be due to the destruction of cellulose glycoside bonds in CNC with the increased of temperature, and the formation of SiO_2_ during thermal degradation of silicon elements in AEAPMDS-CNC prevented its further degradation [22]. This also indirectly proved that AEAPMDS had been successfully grafted onto the cellulose chain of CNC. According to the DTG curve, the maximum degradation rate of CNC appeared at 280 °C, while that of AEAPMDS-CNC arose at 320 °C, which also proved that the thermal stability of AEAPMDS-CNC was better than that of CNC. Therefore, because of the excellent thermal stability of AEAPMDS-CNC, we adopted hot-pressing technology to cover AEAPMDS-CNC formaldehyde-free decorative paper on plywood at 120 °C and the CO_2_ adsorption performance of AEAPMDS-CNC will not be destroyed by temperature.

### 3.5. Analysis of Surface Morphology

**Figure 6:**
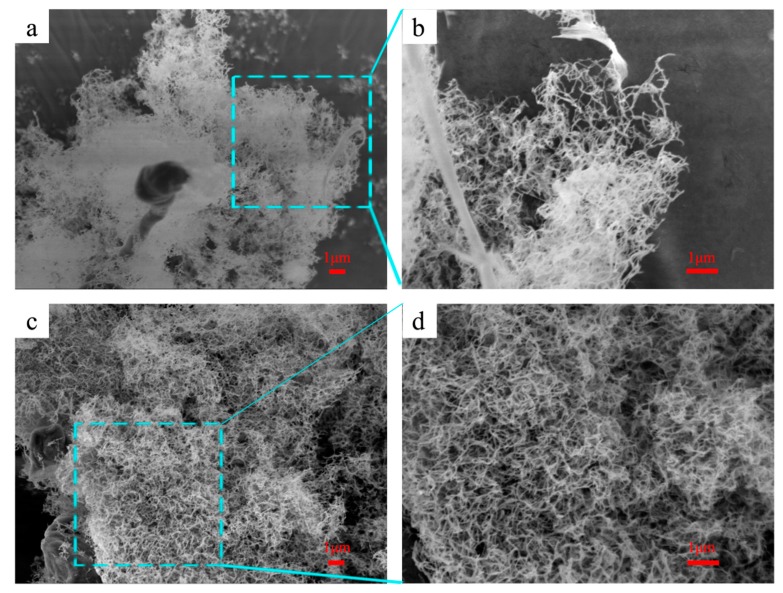
SEM images of CNC (**a**,**b**) and AEAPMDS-CNC (**c**,**d**).

Figure 6 shows the microscopic morphology of CNC and AEPMDS-CNC observed by SEM. The figure shows that AEAPMDS as a modifier has almost no obvious influence on the shape of CNC. Figure 6a,b show that CNC hydrogel has a three-dimensional network structure formed by a series of nanofibers cross-linked with each other through hydrogen bonds. It can be seen from the Figure 6c,d that the morphology of CNC modified by AEAPMDS is continuous and irregular, showing a structure similar to “soft sponge” [23]. This indicated that the chemical reaction between AEAPMDS and CNC did not destroy the porous grid structure of CNC. The existence of a porous structure also provided the basic conditions for AEAPMDS-CNC to be used as a CO_2_ adsorbent. The formation of tert-butanol ice and the addition of the amination agent modified the porous structure of the CNC. The addition of the amination agent was to adjust the capillary skeleton force of the gel skeleton by increasing the contact angle between the gel skeleton and the solvent, then orderly arrange the honeycomb polygonal pore structure in the crystalline amino cellulose crystal. When the amino groups were well embedded on the surface of the CNC, these embedded amino groups reacted with CO_2_, which can improve the adsorption efficiency of the CNC for CO_2_.

### 3.6. Adsorption Performance of the AEAPMDS-CNC Formaldehyde-Free Decorative Paper

**Figure 7:**
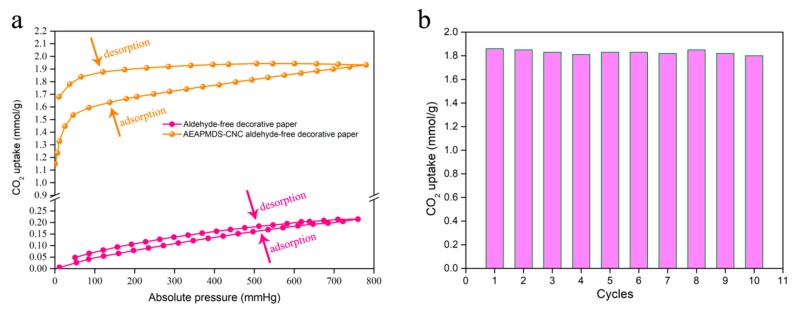
(**a**) The CO_2_ adsorption and desorption isotherms of formaldehyde-free decorative paper grafted with AEAPMDS-CNC at 1 bar under 25 °C; (**b**) the cycling absorption of AEAPMDS-CNC formaldehyde-free decorative paper for 10 times.

We obtained AEAPMDS-CNC formaldehyde-free decorative paper with high CO_2_ adsorption performance under optimal preparation conditions. The CO_2_ adsorption and desorption capacity of the formaldehyde-free decorative paper before and after modification of AEAPMDS-CNC was investigated by measuring the CO_2_ adsorption isotherms at 1 bar and 25 °C (Figure 7a). It can be seen from the figure that the amount of CO_2_ adsorbed by the AEAPMDS-CNC formaldehyde-free decorative paper is significantly increased. Under the condition of 25 °C and atmospheric pressure (760 mmHg), the adsorption capacity of formaldehyde-free decorative paper on CO_2_ was only 0.21 mmol·g^−1^, while the adsorption capacity of CO_2_ after AEAPMDS-CNC modification could reach 1.94 mmol·g^−1^, which increased by 9.24 times. This result was due to the fact that the adsorption of CO_2_ by formaldehyde-free decorative paper without AEAPMDS-CNC modification mainly depended on the cohesion between capillaries of paper fibers on the surface. However, the presence of diaminoids in AEAPMDS-CNC formaldehyde-free decorative paper played an important role on CO_2_ adsorption, which was achieved by chemical adsorption and capillary coagulation [41]. It can be seen from the curve that the adsorption capacity of CO_2_ increased gradually with the promoted of pressure, and the adsorption curve showed a slow upward tendency. At the low pressure stage with the pressure less than 150 mmHg, the CO_2_ adsorption capacity of AEAPMDS-CNC formaldehyde-free decorative paper increased sharply. As the pressure continued to increase, the adsorption curve was consistent with that of formaldehyde-free decorative paper showed a slow increase trend. When AEAPMDS-CNC formaldehyde-free decorative paper was at low pressure, a large amount of CO_2_ had a strong chemical reaction with amino groups on the surface, so a large amount of CO_2_ was captured and the reaction rate increased rapidly. With the increased of relative pressure, the reaction with diamino groups was blocked because of the coating of CO_2_ molecules on the surface. The subsequent reaction depended on the physical adsorption of van der Waals force between molecules, the adsorption capacity was almost linear with the adsorption pressure. It have been investigated that physical adsorption would produce a certain amount of adsorption under pressure more than 7.5 mmHg [42], while AEAPMDS-CNC formaldehyde-free decorative paper will produce a certain amount of adsorption under very low pressure. This also indicated that AEAPMDS-CNC was successfully grafted onto formaldehyde-free decorative paper to give it chemical adsorption properties.

Reusability is an important index for testing adsorption materials. The samples of AEAPMDS-CNC formaldehyde-free decorative paper prepared under the optimum process conditions were adsorbed and desorbed many times to check whether the material had the ability of reuse [43]. The data of 10 adsorption-desorption cycles for AEAPMDS-CNC formaldehyde-free decorative paper samples obtained under the optimum process were tested, as shown in Figure 7b. It can be seen from the figure that after ten cycles of testing, the CO_2_ adsorption performance of the sample was basically stable at 1.8 mmol/g, and the sample showed superior reusability, which was very important for the application of formaldehyde-free decorative paper in the field of adsorbents. Carbamate produced by the reaction of amino with CO_2_ was easy to decompose in a small amount under vacuum, which resulted in a slight decrease in CO_2_ adsorption.

### 3.7. Surface Bond Strength and Abrasion Resistance of the AEAPMDS-CNC Formaldehyde-Free Decorative Paper

**Figure 8:**
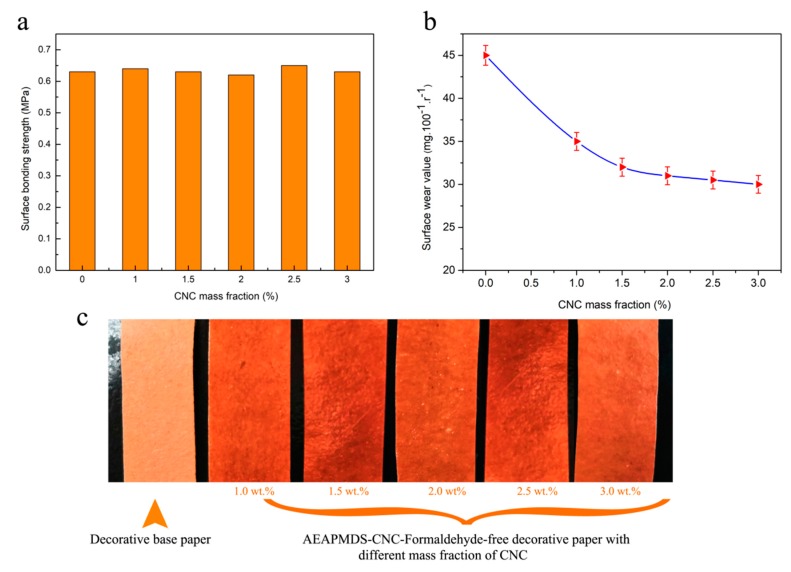
Effect of CNC mass fraction on surface bond strength (**a**) and abrasion resistance (**b**) of AEAPMDS-CNC formaldehyde-free decorative paper. (**c**) Decorative base paper and AEAMMDS-CNC formaldehyde-free decorative paper with different mass fractions of CNC.

It is well known that CNC has a crystal effect as a nano polymer material. It was necessary to study the effect of the amount of CNC added to AEAPMDS-CNC on the surface bond strength and abrasion resistance of the formaldehyde-free decorative paper coating. Figure 8a,b shows the surface bond strength and abrasion resistance of AEAPMDS-CNC formaldehyde-free decorative paper with different mass fractions of CNC. Figure 8c shows the images of decorative base paper and AEAMMDS-CNC-Formaldehyde-free decorative paper with different mass fraction of CNC. According to Figure 8a, it can be seen that the surface bond strength of formaldehyde-free decorative paper before and after modification basically remained at 0.65 MPa, and there was no significant change in the surface bond strength with the addition of CNC mass fraction. This result could be due to the fact that after a certain amount of AEAPMDS-CNC was applied on the formaldehyde-free decorative paper, the water-based acrylic resin adhesive layer on the surface prevented AEAPMDS-CNC from penetrating, so that the surface bond strength of formaldehyde-free decorative paper was not affected by AEAPMDS-CNC. Moreover, as nano materials, CNC has high reactivity and large specific surface area. With the continuous increase of the content, it was easy to self-agglomerate, so it had no effect on the surface bond strength.

The abrasion value refers to the value of paint film before and after being abraded. The smaller the abrasion value, the better the abrasion resistance. The effect of CNC addition on the abrasion resistance of AEAPMDS-CNC formaldehyde-free decorative paper was measured, as shown in Figure 8b. The result showed that the abrasion resistance of formaldehyde-free decorative paper was improved with the addition of CNC concentration in AEAPMDS-CNC, which may be due to the formation of a thin CNC protective layer on the surface of the modified formaldehyde-free decorative paper. Moreover, the existence of Si elements in AEAPMDS also played a decisive role in improving the abrasion resistance of formaldehyde-free decorative paper.

## 4. Conclusions

In this study, AEAPMDS-CNC composite materials were prepared under aqueous heating and coated on formaldehyde-free decorative paper to obtain AEAPMDS-CNC formaldehyde-free decorative paper with high adsorption performance to CO_2_. Upon testing, AEAPMDS was successfully grafted onto the CNC chain without destroying the basic structure of the CNC. By adjusting the addition of the AEAPMDS and CNC, changing the reaction time and temperature, the adsorption of CO_2_ on formaldehyde-free decorative paper could be improved. The adsorption capacity of AEAPMDS-CNC formaldehyde-free decorative paper obtained under the optimum preparation process can reach up to 1.806 mmol/g, and had excellent surface bond strength and abrasion resistance. This work demonstrated the potential of amine-modified CNC in the application of formaldehyde-free decorative paper, and provided a new opportunity for the development of new formaldehyde-free decorative paper. The application of formaldehyde-free decorative paper with high CO_2_ adsorption to plywood and other furniture fields cannot only improve the added value of furniture products but also help protect the indoor environment.
